# Variation in Glucosinolate Accumulation among Different Sprout and Seedling Stages of Broccoli (*Brassica oleracea* var. *italica*)

**DOI:** 10.3390/plants11121563

**Published:** 2022-06-14

**Authors:** Haiyan Lin, Jiayi Sun, Zhiwei Hu, Chenxi Cheng, Sue Lin, Huixi Zou, Xiufeng Yan

**Affiliations:** National and Local Joint Engineering Research Center of Ecological Treatment Technology for Urban Water Pollution, Zhejiang Provincial Key Laboratory for Water Environment and Marine Biological Resources Protection, College of Life and Environmental Science, Wenzhou University, Zhong-Xin Street, Wenzhou 325035, China; 194611372155@stu.wzu.edu.cn (H.L.); plusonesun@163.com (J.S.); huzhiwei.haerbin@aliyun.com (Z.H.); 20451334040@stu.wzu.edu.cn (C.C.); iamkari@163.com (S.L.); zjuzhx@wzu.edu.cn (H.Z.)

**Keywords:** broccoli, cultivar, glucosinolate, quantification, development

## Abstract

Glucosinolates (GLs) are plant secondary metabolites that may act against different types of cancers. Broccoli (*Brassica oleracea* var. *italica*) is rich in GLs which makes it an excellent source of these nutraceuticals. The composition and concentration of GLs vary among broccoli cultivars and throughout the developmental stages of the plant. To obtain the GL profiles of broccoli, GL compositions and contents in four early developmental stages (seeds, 3-day sprouts, 11-day and 17-day seedlings) were determined for nine cultivars of broccoli in this study. A total of 12 GLs including 9 aliphatic GLs and 3 indole GLs were identified from the nine broccoli cultivars using LC-QTOF-MS. UPLC results showed that aliphatic GLs concentrations decreased with broccoli sprouts and seedling growth for most cultivars. Interestingly, indole GLs amounts increased after germination and reached the highest level in 3-day sprouts or 11-day seedlings, and they fell back to a low level in 17-day seedlings. The GL profiles of nine cultivars documented in this study will provide useful information for high quality germplasm selection for cultivation or genetic engineering, and further understanding of the GL metabolic pathways.

## 1. Introduction

Diets rich in Brassicaceae vegetables such as kale, radish, cauliflower, cabbage, and broccoli (*Brassica oleracea* var. *italica*) may reduce the risk of various cardiovascular diseases and cancers [1]. It is mainly attributed to an important class of secondary metabolites in these plants known as glucosinolates (GLs) [2].

The GLs are a group of natural products containing nitrogen and sulfur, mainly found in Brassicaceae plants and a few other families of the Capparales order [3]. In total, 156 GLs have been identified in plants according to the latest report [4]. GLs share a core structure consisting of a β-D-glucopyranose, a (*Z*)-N-hydroximino sulfate ester, and a variable R group [5]. GLs can be classified into aliphatic, indole, and aromatic GLs based on the variable R groups, which are derived from different amino acids [5].

GLs and their derivatives have various biological functions such as anti-oxidative, anti-fungal, anti-bacterial, anti-cancer, and allelopathic properties [6]. The most promising anti-cancer GL derivative is sulforaphane (SF), an isothiocyanates hydrolyzed from glucoraphanin (GRA). It has strong activity against several types of cancers such as prostate, lung, breast, stomach, leukemia, colon, and gastric [7,8,9,10,11]. Additionally, SF may reduce hepatic glucose production, mitigate obesity, and improve glucose control in patients with type II diabetes [12]. Some indole GLs and their hydrolyzates, in particular indole-3-carbinol, have shown anti-carcinogenic and anti-oxidation effects in experimental animals and cultured cells [13].

Brassicaceae sprouts such as broccoli, cabbage, cauliflower, arugula, and radishes are considered to be good sources of GLs [1,14]. Among *Brassica* plants, broccoli may act against different types of cancers because of its high accumulation of GLs, especially GRA [15]. In broccoli, the most abundant GL GRA, which is a precursor of bioactive SF, accounts for over 50% of total GLs [16].

GL profiles vary greatly in different species, cultivars, and throughout the developmental stages [17]. Although 23 different GLs have been identified in *Arabidopsis thaliana*, most species contain less than one dozen GLs [18]. A total of 11 different GLs were detected and monitored in the turnip tubers, through both intact and desulfo-GLs analysis methods [19]. Of the nine *Brassica* crops examined in a previous work reported by Bhandari et al., broccoli reached the highest total GLs concentration in 9-day sprouts (162.19 µmol/g DW) while leaf mustard exhibited the highest total GLs concentration in shoots (61.76 µmol/g DW) and roots (73.61 µmol/g DW) [20]. In the cabbage heads of 146 genotypes, a total of 12 GLs were identified and glucobrassicin (GBS) was the most dominant GL, ranging from 0.79 to 13.14 µmol/g while the GRA content only contributed 0.62 µmol/g [21]. There were 16 GLs identified including 11 aliphatic GLs, 4 aromatic GLs, and an indole GL in broccoli florets of 3 commercial cultivars and 11 inbred broccoli lines by Jo et al. The contents of the total GLs differed much among cultivars—ranging from 7.06 to 22.97 μmol/g DW [22]. The SF yields of broccoli seeds and sprouts among six cultivars were significantly different [23]. SF yields of seeds were significantly higher than that of sprouts for six broccoli cultivars, ranging from 4.47 to 13.19-fold [24]. 

The GLs were reported to decrease with sprout growth in *Brassica* crops [25]. Broccoli exhibited the highest total GL concentration in seeds and sprouts rather than in mature shoots and roots of the nine *Brassica* crops [20]. In broccoli, the accumulation patterns of aliphatic GLs such as GRA, NAP, glucoerucin (GER) and total GLs are similar to other *Brassica* crops which is to decrease with sprout growth [26]. Martinez-Villaluenga et al. reported that indole GLs including GBS, 4-methoxyglucobrassicin (4MGBS), and neoglucobrassicin (NGBS) increased gradually within 5 days of the germination in broccoli [16]. Indole GLs mainly accumulated in roots according to a report in *Arabidopsis thaliana* [27]. However, the patterns of indole GLs such as GBS, 4MGBS, and NGBS in Brassicaceae plants especially in broccoli and their mechanism are unclear during sprout germination and plant growth.

The ultimate application of GLs studies is to specifically alter the quantity and quality of GLs to increase nutrition values and plant protection for specific needs [28]. Chavadej et al. demonstrated that altering a tryptophan decarboxylase (TDC) gene of *Brassica napus* cv. Westar, results in plants that divert tryptophan, the key metabolite, into tryptamine rather than into indole GLs [29]. Effectively, the indole GLs in mature seeds of transgenic plants reduced to 3% of that in non-transformed ones [29].

GLs differ among species, cultivars, and stages of development; thus, a database of GLs of broccoli needs to be built to provide information for selection of cultivar and subsequent cultivate of broccoli with high quality germplasm. In this study, GLs in nine broccoli cultivars (BY: Biyu; WX: Wenxing; YX: Youxiu; LJ80: Lvjian80; LB: Lvbao80; CQJL: Chunqiujiali; LJ100: Lvjian100; ML: Meilv; HJLFS: Huangjinlvfushi) were identified by liquid chromatography–quadrupole time of flight mass spectrometry (LC-QTOF-MS), and their concentrations were monitored by ultra-performance liquid chromatography (UPLC) at four early developmental stages. The documented GL profiles will provide valuable information for further selection of high-quality germplasm for cultivation or genetic engineering.

## 2. Results

### 2.1. Identification of GLs

**Figure 1:**
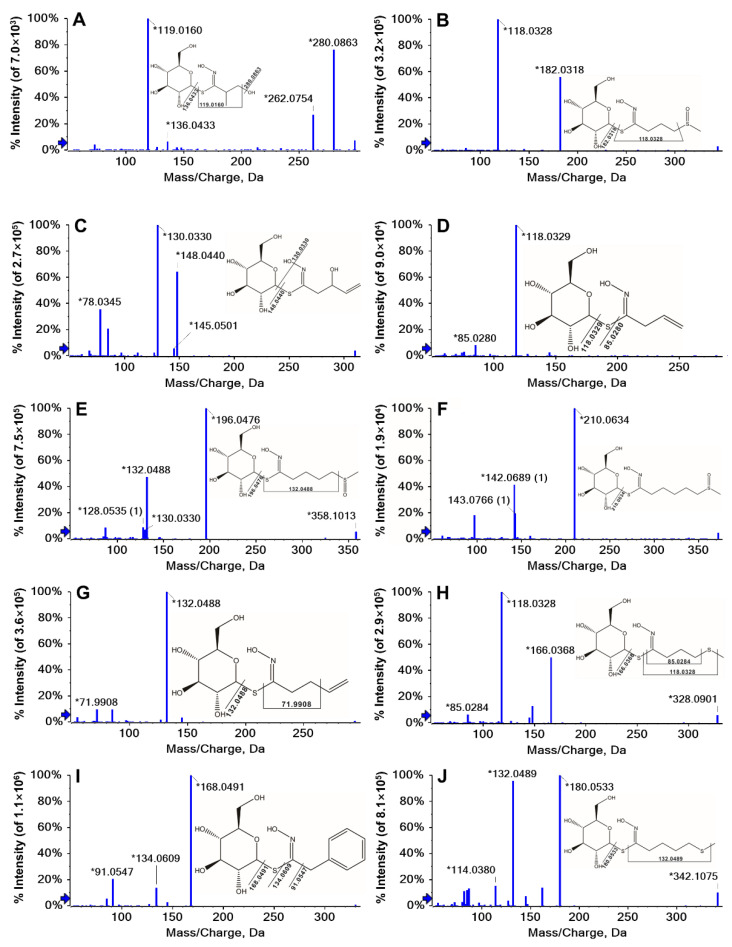
LC–MS/MS spectra of aliphatic glucosinolates and their chemical structures in broccoli. GTP is the internal standard which is an aromatic GL. (**A**), Glucosisymbrin (GSI); (**B**), Glucoiberin (GIB); (**C**), Progoitrin (PRO); (**D**), Sinigrin (SIN); (**E**), Glucoraphanin (GRA); (**F**), Glucoalyssin (GAL); (**G**), Gluconapin (NAP); (**H**), Glucoibervirin (GIV); (**I**), Glucotropaeolin (GTP); (**J**), Glucoerucin (GER). The * sign indicates that this fragment ion is generated by this molecular ion peak, which is judged by the liquid mass software.

**Figure 2:**
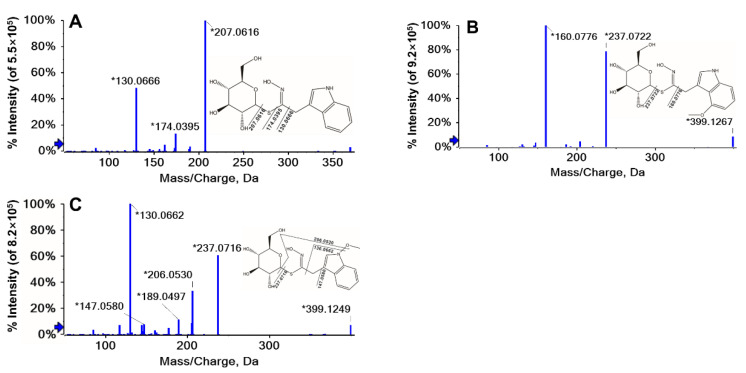
LC–MS/MS spectra of indole glucosinolates and their chemical structures in broccoli. (**A**), Glucobrassicin (GBS); (**B**), 4-Methoxyglucobrassicin (4MGBS); (**C**), Neoglucobrassicin (NGBS). The * sign indicates that this fragment ion is generated by this molecular ion peak, which is judged by the liquid mass software.

GLs were extracted and purified on a DEAE-Sephadex A-25 anion-exchange column followed by conversion to desulfo-GLs. Desulfo-GLs were identified using a tandem mass spectrometer (MS) on a Triple TOFTM 6600 system (Sciex, CA, USA). The identification of desulfo-GLs was performed according to the following criteria: (i) the exact mass analysis of precursor ions is < 5 ppm mass error, (ii) the unique peaks in the treated sample are compared to the blank samples, (iii) there must be at least ≥1 characteristic fragments ion. The retention time (t_R_), chemical molecular formula, accurate mass, and fragments of the desulfo-GLs obtained using LC-QTOF-MS are summarized in Table 1. The chemical structures of the identified GLs including internal standard Glucotropaeolin (GTP) are shown in Figure 1 (aliphatic GLs) and Figure 2 (indole GLs).

A total of 12 GLs were identified from seeds, sprouts, and seedlings of nine broccoli cultivars in this study (Table 1). UPLC chromatograms of the GLs in sprouts of broccoli are given in Figure S1. Nine of the detected GLs were aliphatic GLs including Glucosisymbrin (GSI), Glucoiberin (GIB), Progoitrin (PRO), Sinigrin (SIN), Glucoraphanin (GRA), Glucoalyssin (GAL), Gluconapin (NAP), Glucoibevirin (GIV), and Glucoerucin (GER). The R group of these aliphatic GLs was mainly derived from methionine with different extents of chain-elongations. The length of aliphatic side chain varied from 3 to 5 carbons among the identified GLs. The side chains of GSI, GIB, SIN, and GIV are three-carbon aliphatic chains. PRO, GRA, NAP, and GER have 4-carbon R groups and GAL has a 5-carbon R group. There is a sulfur atom in the R group of GIB, GRA, GAL, GIV, and GER. Moreover, PRO, SIN, and NAP have the same functional group Ethylenic bond. Among them, NAP was easily degraded over time when stored at 4 °C while other GLs with an Ethylenic bond stayed stable (data not shown).

Three indole GLs, Glucobrassicin (GBS), 4-Methoxyglucobrassicin (4MGBS), and Neoglucobrassicin (NGBS), were identified and no aromatic glucosinolate was detected in the current study (Table 1). The structures of the three indole GLs (GBS, 4MGBS, and NGBS) identified in our work are similar, the only difference is whether there is a methoxyl group or at which position the methoxy group is located (Figure 2). There is no methoxy group in GBS, while there is a methoxy in the fourth carbon of benzene in 4MGBS and a methoxy in the nitrogen atom of indole in NGBS.

Three indole GLs, Glucobrassicin (GBS), Methoxyglucobrassicin (4MGBS), and Neoglucobrassicin (NGBS), were identified and no aromatic glucosinolate was detected in the current study (Table 1). The structures of the three indole GLs (GBS, 4MGBS, and NGBS) identified in our work are similar, the only difference is whether there is a methoxyl group or at which position the methoxy group is located (Figure 2). There is no methoxy group in GBS, while there is a methoxy in the fourth carbon of benzene in 4MGBS and a methoxy in the nitrogen atom of indole in NGBS.

Nine broccoli cultivars (BY, WX, YX, LJ80, LB, CQJL, LJ100, ML, and HJLFS) showed different GLs profiles both in compositions and contents. Additional data and details are given in Table S1. Twelve GLs were detected in almost all studied broccoli cultivars except for CQJL. SIN was absent in the CQJL cultivar for all developmental stages. The identified GLs in this work are most abundant in seeds for most cultivars. In 17-day seedlings, some GLs identified in seeds dropped to an undetected level for several cultivars. For example, SIN could not be detected in the 11-day seedlings of WX, YX, LJ80, and LJ100 broccoli. GER disappeared in 11-day seedlings of cultivars BY, WX, YX, LJ80, LB, LJ100, and HJLFS. GIV became absent from 11-day seedlings of BY and WX, while PRO was only absent in YX for seeds, 3-day sprouts, and 17-day seedlings.

### 2.2. Quantification of GLs

**Figure 3:**
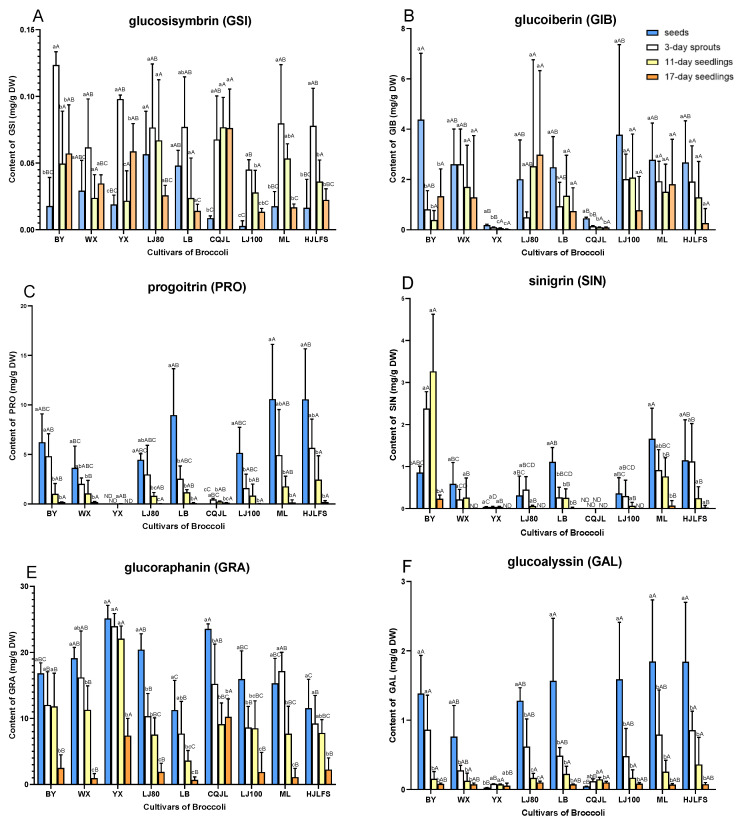

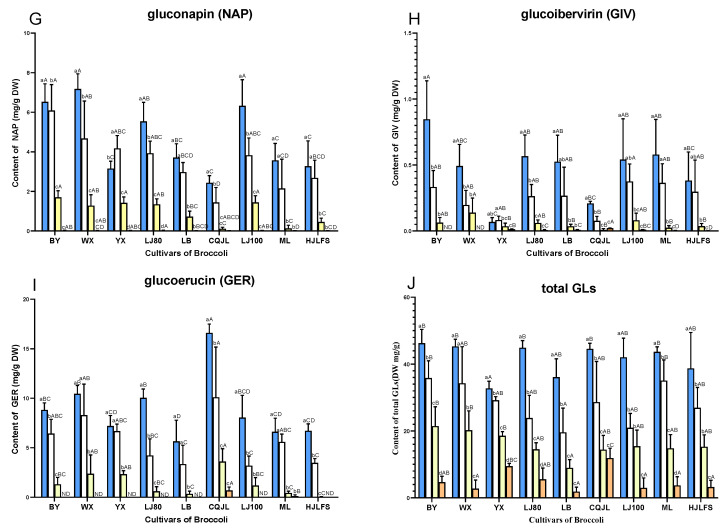
Contents of aliphatic GLs (**A**–**I**) and total GLs (**J**) in different cultivars of broccoli at various developmental stages. (**A**), Glucosisymbrin (GSI); (**B**), Glucoiberin (GIB); (**C**), Progoitrin (PRO); (**D**), Sinigrin (SIN); (**E**), Glucoraphanin (GRA); (**F**), Glucoalyssin (GAL); (**G**), Gluconapin (NAP); (**H**), Glucoibervirin (GIV); (**I**), Glucoerucin (GER); (**J**), total GLs. The different lowercase letters indicated significant differences (*p* < 0.05) of values in different stages in the same cultivar. The different uppercase letters indicated significant differences (*p* < 0.05) of values in cultivars in the same developmental stage. Values are expressed as mean ± standard deviation (SD) (n = 5).

The total GL levels were monitored in broccoli seeds, sprouts, 11-day and 17-day seedlings of the nine cultivars. The total GL concentrations were highest in seeds and they decreased dramatically with aging during the early developmental phases of all cultivars (Figure 3J). Accumulation of the total GLs varied from 36.108 ± 5.491 to 46.251 ± 4.125 mg/g DW in seeds with no significant difference (*p* < 0.05) for most cultivars except for YX. YX seeds had 32.737 ± 2.126 mg/g DW GLs which was significantly (*p* < 0.05) lower than GLs in seeds of other cultivars (Figure 3J).

Among GLs detected in this work, the most abundant glucosinolate was GRA, an aliphatic glucosinolate with health-beneficial functions, for the nine broccoli cultivars. It was accumulated highest in seeds compared to spouts and seedlings. The GRA levels in seeds varied among different cultivars. GRA accumulated in relatively high levels in YX, CQJL, LJ80, and WX seeds and in low levels in LB and HJLFS seeds (Figure 3). GRA concentration showed significant decrease (*p* < 0.05) in 3-day sprouts of LJ80, CQJL, and LJ100 broccoli compared to seeds, in 11-day seedling for WX, LB, ML, and HJLFS compared to 3-day sprouts, and in 17-day seedlings for BY and YX compared to 11-day. GRA reached the lowest level for all 9 cultivars in 17-day seedlings. The largest decrease from seeds to 17-day seedlings was obtained in LJ80 cultivar which was 18.508 ± 1.111 mg/g DW.

GER, which is the precursor of another promising cancer chem-preventive agent erucin, was the second most abundant aliphatic GL. GER concentrations ranged from 5.646 ± 2.140 mg/g DW in LB seeds to 16.602 ± 0.898 mg/g DW in CQJL seeds (Figure 3I). GER levels decreased with plant development and was almost completely consumed after 17 days growing from germination for most cultivars except for CQJL and ML. GER dropped to 0.690 ± 0.346 mg/g DW in 17-day seedlings of CQJL and 0.114 ± 0.135 mg/g DW in 17-day seedlings of ML. The largest decrease in GER from seeds to 17-day seedling was observed in CQJL, which was 15.912 ± 0.552 mg/g DW.

Accumulation of NAP and PRO, another two aliphatic GLs with 4-carbon side chains, was lower than GRA and GER but higher than other detected aliphatic GLs with 3-carbon and 5-carbon side chains in most broccoli cultivars. PRO was not detected in seeds of YX cultivar (Figure 3C). Both NAP and PRO showed decreasing trends as GRA and GER did during plant development. The highest NAP level (7.179 ± 0.764 mg/g DW) and the highest PRO amount 10.579 ± 5.539 mg/g DW were achieved in WX seeds and HJLFS seeds, respectively (Figure 3C,G). Notably, PRO concentration in CQJL was much lower than other cultivars, so the decrease in PRO during development could be negligible in CQJL. In 17-day seedlings of the remaining cultivars, NAP and PRO dropped to trace amounts.

GAL is the only 5-carbon aliphatic GL identified in nine broccoli cultivars in this study. The decrease in GAL during sprout growth was observed for most cultivars except for YX and CQJL. GAL did not show a clear trend with development in YX and CQJL cultivars, which may be due to the very low accumulation of GAL in them. YX and CQJL only had 0.026 ± 0.003 mg/g DW and 0.044 ± 0.003 mg/g DW GAL, respectively, in seeds (Figure 3F). The GAL content did not change significantly (*p* < 0.05) in seeds of the remaining cultivars including BY, WX, LJ80, LB, LJ100, ML, and HJLFS. ML seeds had the highest amount of GAL which was 1.845 ± 0.891 mg/g DW.

GIB, SIN, GIV, and GSI, which belong to aliphatic GLs with 3-carbon aliphatic side chains, were less abundant than aliphatic GLs with 4-carbon side chains for all nine broccoli cultivars (Figure 3). GSI was the least abundant GL (0.009 ± 0.002–0.124 ± 0.010 mg/g DW) among all GLs for 9 different cultivars. Interestingly, different from the GLs discussed above, the highest GSI was achieved in 3-day sprouts during development instead of in seeds for all cultivars. GSI started decreasing after 3 days of growing and decreased to the lowest level in 17-day seedlings of LJ80, LB, LJ100, ML, and HJLFS. In CQJL, the GSI maintained at the same level from 3-day sprouts until 17-day seedlings. For BY, WX, and YX, the GSI concentration decreased from 3-day sprouts to seedlings, but there was no significant change (*p* < 0.05) in GSI concentrations between 11-day and 17-day seedlings (Figure 3A).

GIV showed clear decreasing trends during development as most other aliphatic GLs showed (Figure 3H). The high GIV level was obtained in BY, WX, LJ80, LB, LJ100, ML seeds while the contents of GIV in 17-day seedlings were low. For example, only 0.001 ± 0.002 mg/g DW GIV was detected in 17-day seedlings of HJLFS. GIB accumulated in very low levels in YX and CQJL compared with other cultivars. Since the deviations of GIB were relatively large, there was no significant change (*p* < 0.05) during the four developmental stages for most cultivars except for BY. BY has the highest GIB in seeds which decreased significantly (*p* < 0.05) to 0.493 ± 0.273, 0.232 ± 0.168, and 0.681 ± 0.781 mg/g DW in 3-day sprouts, 11-day seedlings, and 17-day seedlings, respectively. SIN was absent in CQJL and accumulated in trace amounts in YX. Interestingly, different from other cultivars, SIN in BY showed an increasing trend in the early developmental stages. SIN increased from 0.858 ± 0.147 mg/g DW in seeds to 2.378 ± 0.404 in 3-day seedlings and reached the highest level (3.265 ± 1.362 mg/g DW) in 11-day seedlings of the BY cultivar.

**Figure 4:**
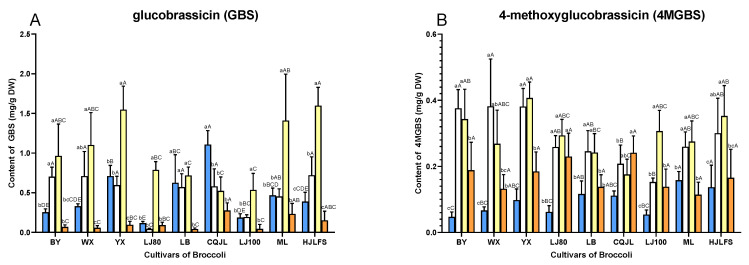

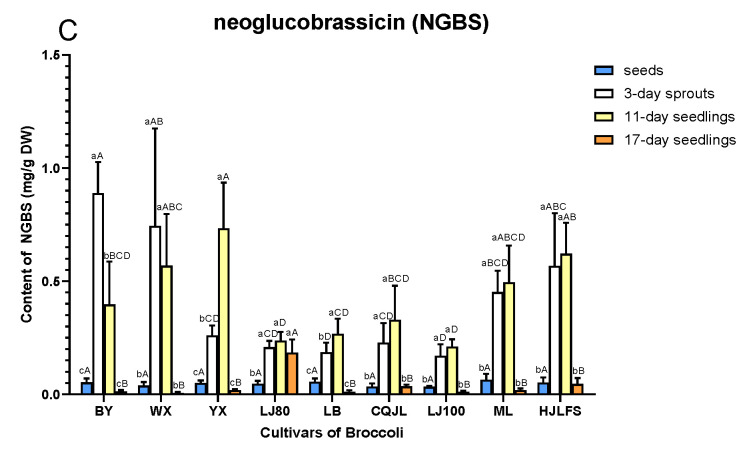
Contents of indole GLs in different cultivars of broccoli at various developmental stages. (**A**), Glucobrassicin (GBS); (**B**), 4-Methoxyglucobrassicin (4MGBS); (**C**), Neoglucobrassicin (NGBS). The different lowercase letters indicated significant differences (*p* < 0.05) in values in different stages in the same cultivar. The different uppercase letters indicated significant differences (*p* < 0.05) in values in cultivars in the same developmental stage. Values are expressed as mean ± standard deviation (SD) (n = 5).

Interestingly, indole GLs showed totally different changing patterns from aliphatic GLs during early development stages which is rarely reported. The contents of indole GLs including GBS, 4MGBS, and NGBS had significantly increased (*p* < 0.05) and reached the highest level in 3-day sprouts or 11-day seedlings compared to the previous stages (Figure 4) and then decreased in 11- or 17-day seedlings. GBS in cultivars BY, WX, YX, LJ80, LJ100, ML, and HJLFS achieved their highest amount in 11-day seedlings and then declined in 17-day seedlings. The high GBS concentrations were obtained in 11-day seedlings of BY, WX, YX, ML, and HJLFS cultivars (Figure 4A). In addition, the contents of GBS in LB did not show a significant change (*p* < 0.05) from seeds to 11-day seedlings, but it decreased sharply in 17-day seedlings. The amount of GBS in CQJL dropped in 3-day sprouts and then stayed relatively stable from 3-day sprouts to 17-day seedlings. 4MGBS in BY, WX, LB, and CQJL cultivars reached the highest level in 3-day sprouts while the levels of 4MGBS in the other cultivars including YX, LJ80, LJ100, ML, and HJLFS reached the highest points in 11-day seedlings (Figure 4B). Among the nine cultivars and different developmental stages, the highest 4MGBS level (0.407 ± 0.048 mg/g DW) was achieved in 11-day seedlings of YX. The amounts of NGBS in cultivars BY and WX reached the maximum in 3-day sprouts while the remaining cultivars reached the maximum in 11-day seedlings. NGBS achieved the highest amount 0.889 ± 0.137 mg/g DW in 3-day sprouts of BY (Figure 4C).

YX and CQJL have significantly (*p* < 0.05) higher GRA contents, which is one of the most beneficial GLs, compared to other cultivars in all stages we studied, especially in seeds. Notably, the total GLs in YX broccoli seeds was the lowest among the nine cultivars, but GRA contributed as high as 76.8% of the total GLs. CQJL seeds were the richest source of GER, another beneficial GL, among different cultivars. The 11-day seedlings of WX, YX, ML, and HJLFS cultivars have significantly higher IGLs (*p* < 0.05) than in other developmental stages and cultivars (Figure 4). Thus, YX would be a good candidate if high GRA and IGLs contents is preferred.

The two-factor test results showed that each cultivar and developmental stage including their interactions had significant effects on the contents of glucosinolates with one exception. The interaction effect of cultivar × developmental stage to GIB was not significant. The results of interactions between tested factors are shown in Table S2.

## 3. Discussion

The composition and content of GLs in *Brassica* plants are affected by environmental factors such as genotype, plant tissue, growth, harvest time, climate, and cultivation conditions. Among them, genotype is usually the main factor affecting glucosinolate composition and content [26,31,32,33].

Profiles of GLs varied in different cultivars and developing stages of the nine cultivars. There were 12 GLs identified and quantified from a total of nine broccoli cultivars in the current work. It was in accordance with previous reports that most species contain a limited number of GLs (usually less than one dozen) [18]. Wang et al. identified seven aliphatic GLs and two indole GLs in Bck1 broccoli seeds from Tokita Seed Co., Ltd., Saitama, Japan [34]. Guo et al. reported seven GLs in “Youxiu” broccoli sprouts including three aliphatic GLs, glucoraphanin (GRA), glucoerucin (GER), and glucoalyssin (GAL), and four indole GLs, 4-hydroxy glucobrassicin (4HGBS), glucobrassicin (GBS), 4-methoxy glucobrassicin (4MGBS), and neoglucobrassicin (NGBS) [35]. Eight aliphatic GLs, four indole GLs, and one aromatic glucosinolate were identified and quantified in broccoli florets by Gu et al. [36]. Compared to their results, two additional aliphatic GLs, which are GSI and GIV, were identified in nine Broccoli cultivars in our work. GSI has been identified in black radish at a very low level [37], but it was seldomly reported in broccoli probably due to the low accumulation in this plant. 4HGBS, gluconasturtiin (NAS), and glucotropaeolin (GTP) were not detected in the broccoli seeds, sprouts, and seedlings because they were rarely detected in *B. oleracea* vegetables [38]. However, NAS and GTP were detected in a relatively low content in broccoli florets by Li et al. while 4HGBS was detected in broccoli seeds by West et al. [38,39]. Jo et al. reported 16 GLs including 11 aliphatic GLs, 4 aromatic GLs, and an indole GL in broccoli florets of 3 commercial cultivars and 11 inbred broccoli lines [22]. Among them, the 4 aromatic GLs are specifically produced in broccoli florets rather than seedlings. The results indicated that most GLs which including PRO, SIN, GRA, GAL, NAP, GER, GBS, 4MGBS, and NGBS widely exist in most broccoli organs, while aromatic GLs such as GTP and NAS are specifically produced in broccoli florets and GSI and GIV are specifically accumulated in seeds, sprouts, and seedlings.

The seeds have the highest stock of aliphatic GLs except for GSI in the current study. As sprouts grow, most aliphatic GLs decreased gradually in all nine cultivars, which is consistent with previous reports [3,26]. It demonstrated that GLs may be consumed as nutrition during the germination and growth of seedlings [40].

The structure of nine identified aliphatic GLs are similar and most of them showed a decreasing trend during early developmental stages of plants. However, the contents of different aliphatic GLs varied a lot. Two important health-beneficial GLs, GRA and GER, were the two top abundant GLs, while other GLs are low in GLs contents especially GSI and GIV. It was in agreement with previous studies that GRA was the most abundant GL in 34 broccoli seed cultivars by West et al. [39].

The similar change patterns of aliphatic GLs except for GSI may be due to the relatively similar biosynthetic and metabolic pathway of aliphatic GLs. In a previous study, Bhandari et al. reported that PRO, GRA, and GER showed significantly positive correlations with each other as well as with total GLs because all of these GSLs are aliphatic GSLs with 4-carbon side chains and follow quite similar biosynthetic pathways [20]. However, the changing pattern of GSI was different from the other eight aliphatic GLs. On one hand, the content of GSI was so low with a highest amount of only 0.1256 mg/g (DW) in 3-day sprouts of BY that the deviations and errors were relatively large (Figure 3). On the other hand, the secondary metabolism mechanism in plants was complex. 

The accumulation of indole GLs over sprouts developing was rarely reported. Indole GLs levels in seeds were generally low according to our results. Wang et al. found that indole GLs levels in broccoli seeds were much lower than those in florets, so broccoli seeds may not be a good resource for indole GLs [34]. Interestingly, indole GL levels increased to the highest level in 3-day sprouts or 11-day seedlings and decreased in 17-day seedlings. It indicated that indole GLs were actively synthesized after germination and during the early development of seedlings. Since plants were transplanted into pots when they were 3-day sprouts, the indole GLs as secondary metabolites may help the plant adapt to the new environment and grow. However, the mechanism and the role of these indole GLs need to be further studied.

## 4. Material and Methods

### 4.1. Chemicals

Sulfatase (from *Helix pomatia*) was purchased from Sigma-Aldrich (St. Louis, MO, USA). The common glucosinolate Glucotropaeolin (Sigma-Aldrich) was used as reference standard for GLs. DEAE Sephadex A-25 was purchased from GE Healthcare (Pittsburgh, PA, USA). Murashige and Skoog Basal (MS) Medium with vitamins was purchased from Phyto Technology Laboratories (Shawnee Mission, KS, USA). Nutrient solution was purchased from The Scotts Miracle-Gro (Marysville, OH, USA). Methanol (HPLC Grade) was purchased from Spectrum Chemical and Laboratory Products (Gardena, CA, USA). Acetic acid (Analytically pure, AR) was purchased from Zhejiang Zhongxing Chemical Reagent Co., Ltd. (Lanxi, Zhejiang, China). Quartz sand (Analytically pure, AR) was purchased from Sinopharm Chemical Reagent Co., Ltd. (Shanghai, China).

### 4.2. Plant Material and Culture Condition

Seeds of nine broccoli cultivars (BY, WX, YX, LJ80, LB, CQJL, LJ100, ML, and HJLFS) were purchased from a local seed company in China. The seeds were sown on 100 mL MS medium with 0.6% agar and kept at 25 °C in the dark for three days [41]. Afterwards, the plants were transplanted into pots containing a mixture of nutrient soil, vermiculite, and perlite (3:1:1, *v*/*v*/*v*) in an artificial climate room for plant cultivation under fluorescent light at 120 μmol/m^2^/s with a 16 h:8 h light:dark cycle at 25 °C and a relative humidity of approximately 60%. The plants were irrigated with water and nutrient solution (N ≥ 30 g/L, P_2_0_5_ ≥ 14 g/L, K_2_O ≥ 16 g/L, Fe ≥ 0.14 g/L, Mn ≥ 0.06 g/L) every three days. They were not invaded by any visible pests.

Broccoli samples were harvested at four early developmental stages: mature seeds (Stage A), 3-day sprouts (Stage B), 11-day seedlings (Stage C), 17-day seedlings (Stage D). Images of broccoli at different developmental stages are given in Figure S2. Stage B was a stage that sprouts grew with no lights in MS Medium and were about to be transplanted into pots. Stage C was a period that seedlings began to grow a main leaf while in Stage D they were growing the second main leaf. Broccoli sprouts at day 3 and seedlings at days 11 and 17 were immediately frozen in liquid nitrogen and stored at −80 °C for further experiments. Each replicate of samples was collected from the same chamber.

### 4.3. Glucosinolate Extraction and Analysis

GLs were extracted, converted to desulfo-GLs, and separated on UPLC as previously described with slight changes [37]. Briefly, freeze-dried samples were ground in 80 °C 70% methanol for 90 s and spiked with Glucotropaeolin (GTP, in 70% methanol, internal standard). The supernatant was collected after centrifugation at 6000× *g* at 4 °C for 10 min. The raffinate was extracted with 70% methanol again. The combined supernatant was purified on a DEAE-Sephadex A-25 anion-exchange column, followed by sulfatase treatment overnight and elution with ultrapure water; the GLs were identified as desulfo-GLs. 

Desulfo-GLs were identified by tandem mass spectrometer (MS) on a Triple TOFTM 6600 system (Sciex, Foster City, CA, USA). Quantification was based on integrative peak areas and normalized against a known amount of an internal standard, benzyl-glucosinolate GTP, which was added at the start of the glucosinolate extraction procedure. The relative response factors of different GLs were also taken into consideration.

### 4.4. LC-MS Conditions

The chromatographic analysis was performed on the Sciex Exion LC system (Foster City, CA, USA) using a reversed phase C18 Waters ACQUITY UPLC HSS T3 column (50 × 2.1 mm, 1.8 μm). The column temperature was maintained at 40 °C. The mobile phase consisted of water (A) and methanol (B). The gradient elution program was described in the following program: 0–7 min, 0–25% B, 7–8.6 min, 25–60% B, 8.6–9.2 min, 60–100% B, 9.2–9.8 min, 100% B, 9.8–10.6 min, 100–0% B, 10.6–13 min, 0% B. The flow rate was set at 0.5 mL/min. Autosampler temperature was set at 15 °C and the injection volume was 5 μL.

All analyses were performed using a quadrupole time-of-flight Triple TOF^TM^ 6600 mass spectrometer (Sciex, Foster City, CA, USA) equipped with a Turbo V™ ion source operating in the positive ESI mode. MS and MS/MS data were collected for each sample using TOF mass spectrometer-information dependent acquisition-enhanced product ion (TOFMS-IDA-EPI) acquisition mode. Data acquisition included a preliminary TOF-MS high-resolution scan (*m*/*z* 100–1000 Da) followed by IDA acquisition using a variable window setup (the 10 most intense ions which form the peak of each acquisition cycle were chosen for a product ion scan at *m*/*z* 50–1000 Da). The optimized MS parameters were set as follows: ion spray voltage, 5500 V; the turbo spray temperature, 550 °C; curtain gas, 35 psi; nebulizer gas (gas 1), 55 psi; heater gas (gas 2), 55 psi; declustering potential, 80 V; collision energy, 35 eV; collision energy spread, 15 eV. Data were acquired using SCIEX OS 1.5 Software. In addition, an automated calibration delivery system (CDS) was used to automatically regulate the MS and MS/MS every five samples.

### 4.5. Statistical Analysis

Independent measurements in five biological replicates were used for each sample in all statistical analyses. The results are presented as the mean ± standard deviation (SD). The data were processed using the GraphPad Prism 8 (San Diego, CA, USA). Statistical analysis was performed using the IBM SPSS Statistics 21 (SPSS Inc., Chicago, IL, USA). Two-way ANOVA and Tukey′s multiple-range tests were used to evaluate the significant differences (*p* < 0.05).

## 5. Conclusions

A total of 12 GLs were identified from nine broccoli cultivars and the accumulation patterns of individual GLs in different early developmental stages of the nine cultivars were described in the present study. Our results indicate that the content and composition of GLs varied among developmental stages and plant genotypes. Aliphatic GL concentrations decreased with seed germination and seedling growth. The relatively high accumulation of GRA and GER in CQJL seeds suggests that CQJL broccoli seeds could be used for the extraction of the beneficial aliphatic GLs in nutraceutical industry. Indole GLs amounts increased after seed germination and reached the highest level in 3-day sprouts or 11-day seedlings, followed by dropping to low levels in 17-day seedlings. The indole GLs changing trend with development has been rarely reported and its physiological meaning needs to be further studied.

## Figures and Tables

**Table 1:** Retention times (t_R_) and MS/MS fragmentation data for the desulfo-GLs of broccoli (*Brassica oleracea* var. *italica*) in positive ionization mode.

No.	t_R_ (min)	Molecular Formula	[M]- *m*/*z* Expt.	[M]- *m*/*z* Calcd.	Error (ppm)	(+) MS/MS *m*/*z* Major Diagnostic ions	Relative Response Factor ^a^	Structure	Identification	Abbr.
1	1.00	C_10_H_19_NO_7_S_1_	298.0955	298.0960	1.9	280.0863, 136.0433, 119.0160	1.39	Aliphatic	Glucosisymbrin	GSI
2	1.49	C_11_H_21_NO_7_S_2_	344.0832	344.0847	4.3	182.0318, 118.0328	1.13	Aliphatic	Glucoiberin	GIB
3	1.81	C_11_H_19_NO_7_S_1_	310.0955	310.0965	3.2	148.0440, 130.0330	1.15	Aliphatic	Progoitrin	PRO
4	2.20	C_10_H_17_NO_6_S_1_	280.0849	280.0858	3	118.0329, 85.0280	1.05	Aliphatic	Sinigrin	SIN
5	2.43	C_12_H_23_NO_7_S_2_	358.0989	358.1008	5.5	196.0476, 132.0488	1.13	Aliphatic	Glucoraphanin	GRA
6	3.55	C_13_H_25_NO_7_S_2_	372.1145	372.1163	4.8	210.0634	1.13	Aliphatic	Glucoalyssin	GAL
7	3.87	C_11_H_19_NO_6_S_1_	294.1006	294.1011	1.8	132.0488, 71.9908	1.17	Aliphatic	Gluconapin	NAP
8	4.15	C_11_H_21_NO_6_S_2_	328.0883	328.0899	4.8	166.0368, 118.0328, 85.0284	1.00	Aliphatic	Glucoibervirin	GIV
9	5.90	C_14_H_19_NO_6_S_1_	330.1006	330.1019	4	168.0491, 134.0609, 91.0547	1.00	Aromatic	Glucotropaeolin ^b^	GTP
10	6.16	C_12_H_23_NO_6_S_2_	342.104	342.1061	6.3	180.0533, 132.0489	1.13	Aliphatic	Glucoerucin	GER
11	6.64	C_16_H_20_N_2_O_6_S_1_	369.1115	369.1136	5.7	207.0616, 174.0395, 130.0666	0.31	Indole	Glucobrassicin	GBS
12	8.41	C_17_H_22_N_2_O_7_S_1_	399.1221	399.124	4.9	237.0722, 160.0776	0.26	Indole	4-Methoxyglucobrassicin	4MGBS
13	9.17	C_17_H_22_N_2_O_7_S_1_	399.1221	399.124	4.9	237.0716, 206.0530, 147.0580, 130.0662	0.21	Indole	Neoglucobrassicin	NGBS

^a^ Thymol-sulfuric acid assay derived UV response factors (relative proportionality factors (RPF)) for desulfo-GLs [30]. ^b^ Glucotropaeolin was added as an internal standard at the start of the extraction procedure. It did not exist in the broccoli according to our study.

## Data Availability

Not applicable.

## Supplementary Materials

The following supporting information can be downloaded at: https://www.mdpi.com/article/10.3390/plants11121563/s1, Table S1: Contents of 12 GLs (mg/g DW) detected in nine cultivars of broccoli; Table S2: Two-way ANOVA results including interactions between factors; Figure S1: UPLC chromatogram of desulfo-GLs identified in HJLFS broccoli seeds. A, Glucosisymbrin (GSI); B, Glucoiberin (GIB); C, Progoitrin (PRO); D, Sinigrin (SIN); E, Glucoraphanin (GRA); F, Glucoalyssin (GAL); G, Gluconapin (NAP); H, Glucoibervirin (GIV); I, Glucotropaeolin (GTP); J, Glucoerucin (GER); K, Glucobrassicin (GBS); L, 4-Methoxyglucobrassicin (4MGBS); M, Neoglucobrassicin (NGBS); Figure S2: Images of broccoli at different developmental stages. **A,** Stage A: seeds; **B,** Stage B: 3-day sprouts; **C,** Stage C: 11-day seedlings; **D,** Stage D: 17-day seedlings.

## Abbreviations

**Abbreviations**	**Full Name**
GLs	glucosinolates
SF	sulforaphane
desulfo-GLs	desulfoglucosinolates
GSI	glucosisymbrin
GIB	glucoiberin
PRO	progoitrin
SIN	sinigrin
GRA	glucoraphanin
GAL	glucoalyssin
NAP	gluconapin
GIV	glucoibervirin
GTP	glucotropaeolin
GER	glucoerucin
GBS	glucobrassicin
4MGBS	4-methoxyglucobrassicin
NGBS	neoglucobrassicin
TDC	tryptophan decarboxylase
BY	Biyu
WX	Wenxing
YX	Youxiu
LJ80	Lvjian80
LB	Lvbao80
CQJL	Chunqiujiali
LJ100	Lvjian100
ML	Meilv
HJLFS	Huangjinlvfushi
UPLC	ultra-performance liquid chromatography
NAS	gluconasturtiin
4HGBS	4-hydroxy glucobrassicin
RPF	relative proportionality factors
DW	dry weight.
LC-QTOF-MS	liquid chromatography-quadrupole time of flight mass spectrometer
