# Supplementary material for: Variation in Glucosinolate Accumulation among Different Sprout and Seedling Stages of Broccoli (Brassica oleracea var. italica)
Source: Plants (Basel). 2022 Jun 14;11(12):1563. doi: 10.3390/plants11121563 (PMC9227298; doi:10.3390/plants11121563)

Table S1. Contents of twelve GLs (mg/g DW) detected in nine cultivars of broccoli.

Cultivar		GSI	GIB	PRO	SIN	GRA	GAL	NAP	GIV	GER	GBS	4MGBS	NGBS
BY	A	0.018±0.021	6.288±0.396	6.238±2.858	0.858±0.147	16.820±1.591	1.383±0.551	6.527±0.910	0.847±0.291	8.827±0.708	0.253±0.044	0.047±0.015	0.053±0.018
	B	0.124±0.010	0.493±0.273	4.812±2.272	2.378±0.404	12.040±5.039	0.862±0.500	6.084±1.311	0.334±0.124	6.430±1.438	0.702±0.119	0.376±0.057	0.889±0.137
	C	0.050±0.039	0.232±0.168	1.018±1.053	3.265±1.362	11.815±5.051	0.153±0.106	1.699±0.334	0.064±0.039	1.307±0.695	0.964±0.402	0.343±0.090	0.398±0.189
	D	0.057±0.036	0.681±0.781	0.186±0.029	0.232±0.088	2.490±2.000	0.081±0.012	0.027±0.008	ND	ND	0.069±0.026	0.188±0.085	0.013±0.006
WX	A	0.029±0.023	2.602±1.403	3.648±2.198	0.590±0.505	19.122±1.635	0.761±0.449	7.179±0.764	0.493±0.163	10.447±0.842	0.329±0.032	0.067±0.011	0.039±0.015
	B	0.062±0.036	0.210±0.109	2.031±0.594	0.218±0.237	16.197±7.058	0.275±0.074	4.678±1.891	0.197±0.110	8.297±3.129	0.710±0.310	0.382±0.143	0.745±0.430
	C	0.024±0.017	0.722±0.924	1.059±1.321	0.259±0.470	11.302±3.640	0.123±0.113	1.282±0.552	0.139±0.111	2.378±1.890	1.102±0.408	0.268±0.102	0.569±0.227
	D	0.035±0.007	0.200±0.339	0.181±0.074	ND	0.965±0.697	0.075±0.018	0.020±0.006	ND	ND	0.057±0.027	0.132±0.043	0.006±0.004
YX	A	0.019±0.007	0.188±0.034	ND	0.038±0.007	25.134±1.999	0.026±0.003	3.152±0.376	0.068±0.034	7.189±1.069	0.709±0.135	0.098±0.034	0.050±0.012
	B	0.098±0.003	0.098±0.013	ND	0.040±0.010	23.955±1.920	0.080±0.004	4.175±0.647	0.083±0.030	6.683±0.710	0.597±0.111	0.381±0.055	0.260±0.044
	C	0.022±0.023	0.062±0.017	0.025±0.017	0.038±0.012	22.064±1.928	0.073±0.008	1.418±0.304	0.036±0.024	2.336±0.343	1.547±0.296	0.407±0.048	0.733±0.203
	D	0.059±0.021	0.028±0.006	ND	ND	7.390±2.630	0.054±0.039	0.023±0.006	0.013±0.003	ND	0.095±0.043	0.185±0.059	0.018±0.005
LJ80	A	0.057±0.032	2.010±1.561	4.459±0.613	0.312±0.461	20.401±2.432	1.279±0.189	5.538±0.964	0.566±0.161	10.045±0.887	0.116±0.022	0.062±0.019	0.047±0.013
	B	0.077±0.048	0.493±0.218	2.987±2.939	0.449±0.312	10.335±3.446	0.618±0.398	3.926±0.615	0.263±0.089	4.219±1.680	0.042±0.009	0.259±0.034	0.209±0.028
	C	0.067±0.046	0.194±0.099	0.817±0.342	0.060±0.018	7.542±2.549	0.166±0.066	1.346±0.281	0.061±0.023	0.604±0.459	0.788±0.104	0.294±0.048	0.237±0.039
	D	0.026±0.008	0.092±0.012	0.011±0.024	ND	1.893±1.321	0.101±0.021	0.032±0.007	0.007±0.005	ND	0.092±0.036	0.230±0.071	0.185±0.057
LB	A	0.048±0.011	2.483±1.221	8.962±4.705	1.111±0.342	11.258±4.502	1.565±0.904	3.713±0.692	0.525±0.201	5.646±2.140	0.625±0.354	0.116±0.040	0.055±0.014
	B	0.077±0.038	0.819±0.840	2.542±1.290	0.266±0.240	7.687±4.896	0.486±0.119	2.973±0.487	0.267±0.217	3.351±1.900	0.570±0.170	0.245±0.063	0.186±0.042
	C	0.024±0.030	0.344±0.487	1.169±0.275	0.253±0.215	3.598±1.556	0.225±0.111	0.724±0.282	0.036±0.015	0.347±0.274	0.717±0.108	0.242±0.057	0.267±0.067
	D	0.014±0.005	0.069±0.120	0.088±0.044	0.012±0.017	0.681±0.4560	0.072±0.008	0.017±0.003	0.008±0.003	ND	0.042±0.013	0.138±0.037	0.012±0.006
CQJL	A	0.009±0.002	0.454±0.048	0.022±0.003	ND	23.560±0.760	0.044±0.003	2.437±0.363	0.209±0.016	16.602±0.898	1.108±0.175	0.111±0.014	0.035±0.013
	B	0.068±0.033	0.141±0.030	0.449±0.121	ND	15.233±6.017	0.119±0.033	1.443±0.754	0.077±0.034	10.099±5.068	0.580±0.220	0.208±0.057	0.229±0.086
	C	0.077±0.022	0.098±0.019	0.240±0.065	ND	9.131±3.203	0.145±0.031	0.113±0.075	0.009±0.009	3.613±1.285	0.523±0.176	0.175±0.047	0.330±0.149

Cultivar		GSI	GIB	PRO	SIN	GRA	GAL	NAP	GIV	GER	GBS	4MGBS	NGBS
	**D**	0.076±0.029	0.089±0.026	0.135±0.014	ND	10.236±2.718	0.100±0.015	0.020±0.002	0.022±0.002	0.690±0.346	0.277±0.096	0.241±0.051	0.035±0.008
**LJ100**	**A**	0.003±0.004	6.048±2.520	5.150±2.586	0.361±0.374	15.960±4.275	1.589±0.821	6.322±1.320	0.542±0.308	8.045±2.257	0.187±0.048	0.054±0.014	0.033±0.004
	**B**	0.045±0.007	2.013±0.994	1.594±1.410	0.296±0.379	8.614±3.222	0.481±0.399	3.824±0.876	0.376±0.133	3.191±0.973	0.193±0.030	0.153±0.012	0.170±0.052
	**C**	0.028±0.017	2.074±1.735	0.834±1.175	0.065±0.082	8.504±4.171	0.168±0.116	1.442±0.341	0.081±0.054	1.188±0.794	0.537±0.207	0.307±0.063	0.211±0.032
	**D**	0.013±0.003	0.183±0.215	0.007±0.017	ND	1.870±2.982	0.085±0.012	0.023±0.005	0.009±0.001	ND	0.044±0.056	0.138±0.054	0.011±0.006
**ML**	**A**	0.018±0.011	3.240±1.198	10.579±5.539	1.663±0.725	15.329±3.767	1.845±0.891	3.570±0.852	0.579±0.267	6.621±1.365	0.466±0.092	0.158±0.027	0.064±0.026
	**B**	0.080±0.044	2.185±0.643	4.930±4.608	0.915±0.487	17.162±2.859	0.791±0.645	2.149±1.483	0.364±0.146	5.591±0.798	0.453±0.096	0.259±0.045	0.452±0.093
	**C**	0.053±0.011	1.572±0.736	1.778±1.033	0.764±0.445	7.676±4.170	0.257±0.164	0.127±0.165	0.024±0.016	0.439±0.187	1.410±0.585	0.275±0.063	0.495±0.163
	**D**	0.017±0.003	0.631±1.093	0.149±0.270	0.071±0.117	1.116±1.329	0.070±0.015	0.010±0.003	0.001±0.003	0.114±0.135	0.232±0.133	0.114±0.038	0.018±0.008
**HJLFS**	**A**	0.016±0.021	3.653±1.369	10.558±5.114	1.149±0.964	11.548±4.389	1.842±0.857	3.277±1.277	0.382±0.216	6.712±0.704	0.388±0.118	0.137±0.067	0.052±0.023
	**B**	0.078±0.028	2.372±1.141	5.661±2.909	1.123±0.902	9.228±4.248	0.855±0.273	2.683±0.894	0.298±0.240	3.475±0.431	0.720±0.232	0.300±0.106	0.568±0.233
	**C**	0.036±0.016	0.570±0.432	2.447±2.420	0.248±0.272	7.796±2.022	0.361±0.392	0.453±0.196	0.037±0.020	0.032±0.035	1.597±0.231	0.353±0.092	0.621±0.137
	**D**	0.022±0.008	0.005±0.010	0.191±0.150	0.022±0.048	2.259±1.766	0.076±0.024	0.012±0.009	0.001±0.002	ND	0.152±0.115	0.166±0.086	0.046±0.026

Values are expressed as mean ± standard deviation (SD) (n = 5), and ″ND″ stands for not detected.

GSI: Glucosisymbrin; GIB: Glucoiberin; PRO: Progoitrin; SIN: Sinigrin; GRA: Glucoraphanin; GAL: Glucoalyssin; NAP: Gluconapin; GIV: Glucoibervirin; GER: Glucoerucin; GBS: Glucobrassicin; MGBS: 4-Methoxyglucobrassicin; NGBS: Neoglucobrassicin.

The broccoli cultivars represented by the abbreviations listed above are as follows. BY: Biyu; WX: Wenxing; YX: Youxiu; LJ80: Lvjian80; LB: Lvbao80; CQJL: Chunqiujiali; LJ100: Lvjian100; ML: Meilv; HJLFS: Huangjinlvfushi.

A, Stage A: seeds; B, Stage B: 3-day sprouts; C, Stage C: 11-day seedlings; D, Stage D: 17-day seedlings.

Table S2. two-way ANOVA results including interactions between factors

source	SS	df	MS	F	P
**dependent variable: GSI**					
developing stages	.075	3	.025	42.648	<0.001
cultivars	.025	8	.003	5.291	<0.001
developing stages × cultivars	.045	24	.002	3.220	<0.001
error	.084	144	.001		
**dependent variable: GIB**					
developing stages	57.953	3	19.318	7.699	<0.001
cultivars	92.082	8	11.510	4.587	<0.001
developing stages × cultivars	73.079	24	3.045	1.214	.240
error	361.328	144	2.509		
**dependent variable: PRO**					
developing stages	761.992	3	253.997	57.873	<0.001
cultivars	431.514	8	53.939	12.290	<0.001
developing stages × cultivars	395.691	24	16.487	3.757	<0.001
error	631.997	144	4.389		
**dependent variable: SIN**					
developing stages	11.789	3	3.930	22.840	<0.001
cultivars	45.219	8	5.652	32.854	<0.001
developing stages × cultivars	34.103	24	1.421	8.259	<0.001
error	24.775	144	.172		
**dependent variable: GRA**					
developing stages	5044.870	3	1681.623	147.553	<0.001
cultivars	2644.903	8	330.613	29.009	<0.001
developing stages × cultivars	890.166	24	37.090	3.254	<0.001
error	1641.127	144	11.397		
**dependent variable: GAL**					
developing stages	31.255	3	10.418	76.862	<0.001
cultivars	11.100	8	1.387	10.236	<0.001
developing stages × cultivars	13.018	24	.542	4.002	<0.001
error	19.519	144	.136		
**dependent variable: NAP**					
developing stages	630.583	3	210.194	404.417	<0.001
cultivars	124.600	8	15.575	29.967	<0.001
developing stages × cultivars	92.027	24	3.834	7.378	<0.001
error	74.844	144	.520		
**dependent variable: GIV**					
developing stages	5.977	3	1.992	120.990	<0.001
cultivars	1.097	8	.137	8.325	<0.001
developing stages × cultivars	1.507	24	.063	3.814	<0.001
error	2.371	144	.016		

**source**	**SS**	**df**	**MS**	**F**	**P**
**dependent variable: GER**					
developing stages	2214.546	3	738.182	384.316	<0.001
cultivars	442.467	8	55.308	28.795	<0.001
developing stages × cultivars	285.317	24	11.888	6.189	<0.001
error	276.590	144	1.921		
**dependent variable: GBS**					
developing stages	18.686	3	6.229	157.787	<0.001
cultivars	5.157	8	.645	16.331	<0.001
developing stages × cultivars	8.262	24	.344	8.721	<0.001
error	5.684	144	.039		
**dependent variable: 4MGBS**					
developing stages	1.241	3	.414	112.142	<0.001
cultivars	.217	8	.027	7.355	<0.001
developing stages × cultivars	.419	24	.017	4.727	<0.001
error	.531	144	.004		
**dependent variable: NGBS**					
developing stages	6.423	3	2.141	156.901	<0.001
cultivars	1.368	8	.171	12.527	<0.001
developing stages × cultivars	2.999	24	.125	9.156	<0.001
error	1.965	144	.014		

SS: mean deviation sum of squares; df: degrees of freedom; MS: mean square.

GSI: Glucosisymbrin; GIB: Glucoiberin; PRO: Progoitrin; SIN: Sinigrin; GRA: Glucoraphanin; GAL: Glucoalyssin; NAP: Gluconapin; GIV: Glucoibervirin; GER: Glucoerucin; GBS: Glucobrassicin; 4MGBS: 4-Methoxyglucobrassicin; NGBS: Neoglucobrassicin.

Figure S1. UPLC chromatogram of desulfo-GLs identified in HJLFS broccoli seeds. A, Glucosisymbrin (GSI); B, Glucoiberin (GIB); C, Progoitrin (PRO); D, Sinigrin (SIN); E, Glucoraphanin (GRA); F, Glucoalyssin (GAL); G, Gluconapin (NAP); H, Glucoibervirin (GIV); I, Glucotropaeolin (GTP); J, Glucoerucin (GER); K, Glucobrassicin (GBS); L, 4-Methoxyglucobrassicin (4MGBS); M, Neoglucobrassicin (NGBS).

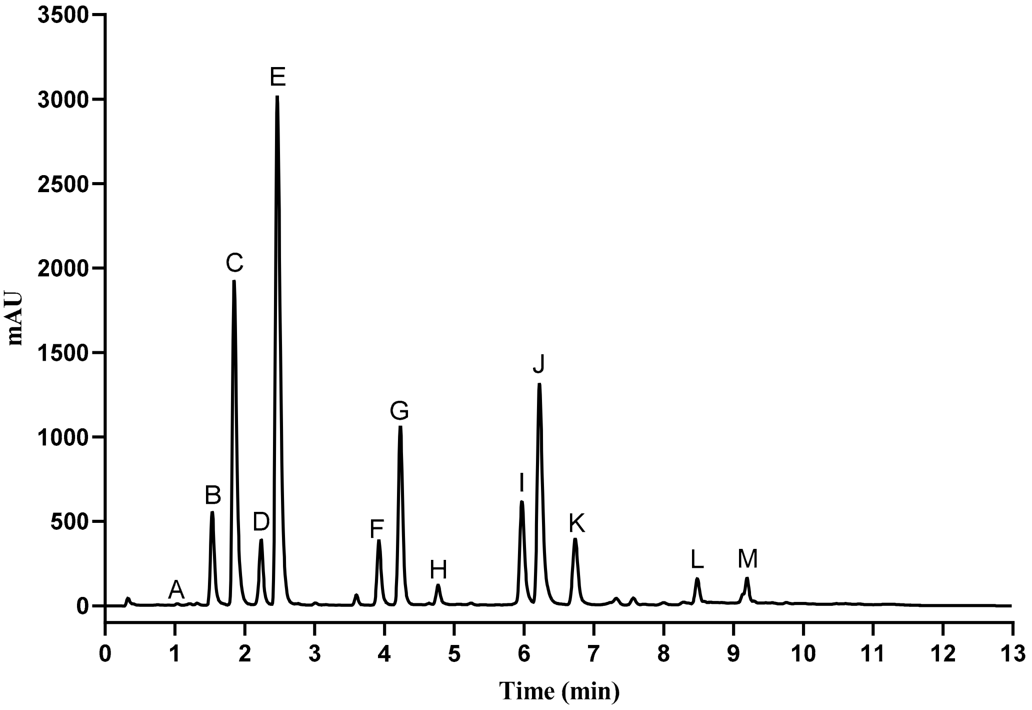

Figure S2. Images of broccoli at different developmental stages. **A,** Stage A: seeds; **B,** Stage B: 3-day sprouts; **C,** Stage C: 11-day seedlings; **D,** Stage D: 17-day seedlings.

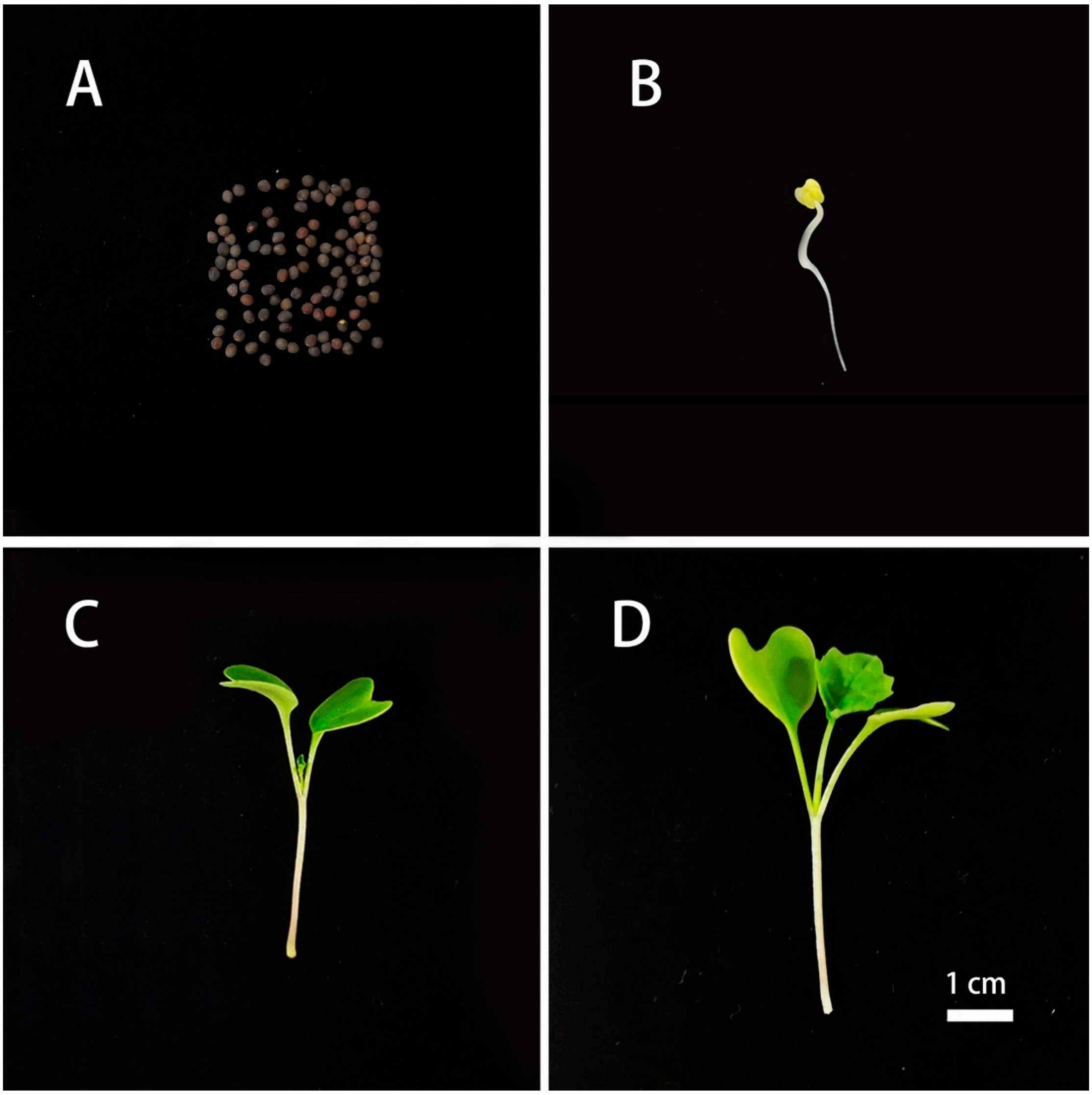

Supplement: Supplementary file 1 [file plants-11-01563-s001.zip › plants-1738302-supplementary.pdf]
